# Analysis of factors associated with excess weight in school children

**DOI:** 10.1016/j.rppede.2016.04.005

**Published:** 2016

**Authors:** Renata Paulino Pinto, Altacílio Aparecido Nunes, Luane Marques de Mello

**Affiliations:** a Programa de Pós-Graduação em Saúde na Comunidade, Faculdade de Medicina de Ribeirão Preto, Universidade de São Paulo (FMRP/USP), Ribeirão Preto, SP, Brazil; b Departamento de Medicina Social, Faculdade de Medicina de Ribeirão Preto, Universidade de São Paulo (FMRP/USP), Ribeirão Preto, SP, Brazil

**Keywords:** Obesity, Overweight, Dietary habits, Health behavior, School children

## Abstract

**Objective:**

To determine the prevalence of overweight and obesity in schoolchildren aged 10 to 16 years and its association with dietary and behavioral factors.

**Methods:**

Cross-sectional study that evaluated 505 adolescents using a structured questionnaire and anthropometric data. The data was analyzed through the T Test for independent samples and Mann-Whitney Test to compare means and medians, respectively, and Chi^2^ Test for proportions. Prevalence ratio (RP) and the 95% confidence interval was used to estimate the degree of association between variables. The logistic regression was employed to adjust the estimates to confounding factors. The significance level of 5% was considered for all analysis.

**Results:**

Excess weight was observed in 30.9% of the schoolchildren: 18.2% of overweight and 12.7% of obesity. There was no association between weight alterations and dietary/behavioral habits in the bivariate and multivariate analyses. However, associations were observed in relation to gender. Daily consumption of sweets [PR=0.75 (0.64-0.88)] and soft drinks [PR=0.82 (0.70-0.97)] was less frequent among boys; having lunch daily was slightly more often reported by boys [OR=1.11 (1.02-1.22)]. Physical activity practice of (≥3 times/week) was more often mentioned by boys and the association measures disclosed two-fold more physical activity in this group [PR=2.04 (1.56-2.67)] when compared to girls. Approximately 30% of boys and 40% of girls stated they did not perform activities requiring energy expenditure during free periods, with boys being 32% less idle than girls [PR=0.68 (0.60-0.76)].

**Conclusions:**

A high prevalence of both overweight and obesity was observed, as well as unhealthy habits in the study population, regardless of the presence of weight alterations. Health promotion strategies in schools should be encouraged, in order to promote healthy habits and behaviors among all students.

## Resumo

**Objetivo:**

Determinar a prevalência de sobrepeso e obesidade em escolares entre 10 e 16 anos e sua associação com fatores alimentares e comportamentais.

**Métodos:**

Este é um estudo transversal, que avaliou 505 escolares através de questionário estruturado e dados de antropometria. Para a análise dos dados foram utilizados o Teste T para amostras independentes e o Teste de Mann-Whitney para comparação de médias e medianas, respectivamente, e o Teste do Chi^2^ para proporções. A Razão de Prevalência (RP) e seus respectivos intervalos de confiança a 95% foi empregado como estimador de associação e regressão logística múltipla para ajustamento por fatores de confusão. Em todas as análises, considerou-se um nível de significância de 5%.

**Resultados:**

Observou-se excesso de peso em 30,9% dos escolares: 18,2% de sobrepeso e 12,7% de obesidade. Não se observou associação entre as alterações de peso e hábitos alimentares/comportamentais nas análises bivariadas e multivariadas. Entretanto, foram encontradas associações quanto ao sexo*.* Consumir doces [RP=0,75 (0,64-0,88)] e refrigerantes diariamente [RP=0,82 (0,70-0,97)] foram hábitos relatados por 273 (54,1%) crianças, menos frequentemente pelos meninos. Almoçar diariamente foi hábito discretamente mais observado entre os meninos [RP 1,11 (1,02-1,22)]. Praticar atividade física (≥3 vezes/semana) foi mais referido pelos meninos e as medidas de associação revelaram duas vezes mais atividade física nesse grupo [RP=2,04 (1,56-2,67)], quando comparado com o das meninas. Cerca de 30% dos meninos e 40% das meninas disseram não realizar atividades com gasto de energia nos períodos livres, sendo os meninos 32% menos ociosos do que as meninas [RP=0,68 (0,60-0,76)].

**Conclusões:**

Observou-se alta prevalência tanto de sobrepeso e obesidade quanto de hábitos pouco saudáveis na população estudada, independentemente da presença de alterações do peso. Estratégias de promoção da saúde nas escolas devem ser estimuladas, no sentido de se incentivar hábitos e comportamentos saudáveis entre todos os estudantes.

## Introduction

Obesity is a chronic disease characterized by excess body fat with consequent health damage.^1^ The World Health Organization (WHO) considers obesity a public health problem associated with high morbidity and mortality rates.^1^ According to the Brazilian Association for the Study of Obesity and Metabolic Syndrome (ABESO), exogenous obesity represents approximately 95% of cases^2^ and, similarly to what occurs in adults, childhood obesity is also accompanied by comorbidities such as systemic arterial hypertension (SAH), glucose intolerance, orthopedic problems and cholesterol metabolism alterations, among others.^2^ Genetic factors and a positive balance between caloric intake and energy expenditure are some of the etiological factors involved in the pathogenesis of obesity.^2^ Among the exogenous factors, overfeeding in the early years of life seems to influence the number of adipocytes, probably explaining why 50%-65% of obese adults were obese children.^3^ Adolescence is considered by some authors one of the most critical periods of human development, during which typical physiological changes seem, among other things, to increase the risk of obesity development and its future persistence.^4^

However, obesity is known to have a multifactorial etiology, where environmental and behavioral factors seem to influence its onset, most likely justifying the variable prevalence observed between different regions. The lowest prevalence rates of obesity in children and adolescents are observed in Asia (2.9%) and Africa (3.9%),^5^^,^ 6 whereas the highest ones are found in the United States (31.8%).^7^ In Latin America, the prevalence of obesity among preschool children is 6.2% in Costa Rica, 6.5% in Bolivia, 7% in Chile and 7.3% in Argentina.^8^ Brazilian Studies in the North and Northeast regions show that overweight and obesity affect 25%-30% of children between five and nine years, whereas in the Southeast and Midwest regions this percentage ranges from 32% to 40%.^9^ Another study in Rio Grande do Sul and Santa Catarina found a prevalence of 14.4% of overweight and 7.5% of obesity in preschool children.^10^

Although the state of São Paulo has the highest population concentration and it is where many studies on this subject are developed, the few studies in the literature involving the population of schoolchildren in preadolescence and adolescence date back to more than 10 years and have prevalence rates that vary considerably.^11^^-^ 14 Additionally, data on obesity/overweight in healthy children who do not undergo medical treatment or supervision and have overweight as the only factor to be investigated, are also scarce.

Therefore, considering that approximately 97% of children and adolescents aged 7-14 years are enrolled in public and private schools^15^ and most of them spend much of their time at school, this study aimed to determine the prevalence and possible risk factors associated with overweight and obesity in schoolchildren aged between 10 and 16 years, in order to quantify the local occurrence of this important problem considered to be of public health concern and establish its association with some common aspects of this population's routine life.

## Method

This is a cross-sectional study, carried out in Elementary Schools in the city of Ribeirão Preto, state of São Paulo, Brazil, involving students aged 10-16 years. The study was approved by Ribeirão Preto School Board (Registration 879/073/2012) and approved by the Institutional Review Board of Hospital das Clínicas da Faculdade de Medicina de Ribeirão Preto (FMRP-USP).

According to data from the state Education Secretariat, 16,785 children and adolescents were enrolled in 2013, in 66 schools of the Ribeirão Preto School Board. To obtain a representative sample of the studied population, two schools were selected from each city district, with a total of 11 schools participating in the study.

To calculate sample size, we considered a prevalence rate of 40% of overweight and obesity among children and adolescents, admitting a maximum sampling error of 5% and a 95% confidence level, according to the formula (finite population):



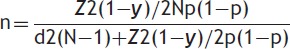



Therefore, a minimum sample size of 361 students was estimated. Anticipating losses and aiming to maintain region representativeness, the total number consisted of 505 students. Only students enrolled in the public elementary schools of Ribeirão Preto school network, who agreed to participate and whose parents or guardians authorized their participation were considered eligible. Pregnant students were excluded, as well as adolescents with diseases that could interfere with body composition.

Data were collected through field research, with visits being scheduled to selected schools and interviews with students carried out over the years 2013 and 2014. During the first visit, five students from each school year/grade were randomly selected and at that time, they received information about the research and the procedures to be carried out at the following visits. The terms of assent and consent were given to each selected child to be taken to the parents. The interviews and anthropometric measurements (weight, height, waist, hip) were performed after the parents or legal guardians gave their permission.

Body mass was measured using a Plenna standard portable digital scale (Plenna^®^, São Paulo, SP, Brazil), certified by INMETRO and that had its own certification (two weighings), with an error margin of±100g. For the measurements of waist and hip circumference, a 2-meter Sanny anthropometric measuring tape (Sanny^®^, São Paulo, SP, Brazil) was used. Height was measured using a portable, retractable WISO 210cm compact stadiometer (WISO^®^, Sao Jose, SC, Brazil), with a frontal display and measurement screen with resolution in millimeters (1mm) and numbered at every centimeter. For better handling and transport of the device, a stainless steel platform was produced on which the stadiometer was set to allow the transport and measurement in a standing and vertical position.

Body Mass Index (BMI) was used to characterize overweight and obesity, calculated according to the WHO recommendations.^1^ The parameters used for the diagnoses were Z-scores of BMI/age, for which percentiles>85 and ≤97 represent overweight;>97 and ≤99.9 represent obesity and percentiles>99.9 correspond to severe obesity, considering reference values for children and adolescents aged 5 to up to 20 years of age.

Data regarding the practice of physical activity, diet and lifestyle were obtained through a structured questionnaire consisting of closed questions, where students answered about their dietary habits (water intake, having breakfast, lunch, snacks between meals and dinner daily; eating sweets and drinking soft drinks) and behavioral habits, such as screen time, including television, computer and video game use, and the type of activities they performed in their free time.

The collected data were stored in Excel spreadsheets 2010^®^ (California, USA) and the entries were made and verified by two people. Data were analyzed using the STATA software, version 13.0 (Texas, USA) and MedCalc^®^, version 13.2.2.1 (Ostend, Belgium). The descriptive analyses of data are shown in tables, containing absolute and relative frequencies (categorical data). As most quantitative data showed an asymmetric distribution in the normality tests, it was decided to show them as medians and their respective interquartile (IQ) intervals. Only the variables age and height are shown as mean and standard deviation.

When analyzing the continuous variables, Student's *t*-test was used for independent samples and the Mann-Whitney test for comparison of means and medians, respectively. The chi-square test was used to compare the proportions between groups. When analyzing the categorical data, prevalence ratio (PR) and their respective 95% confidence intervals were used to estimate the degree of association between variables. The multiple logistic regression method was employed to adjust the estimates for confounding factors. A significance level of 5% was considered in all analyses.

## Results

Of the 505 studied children, 265 (53%) were males and 91% belonged to the age group of 10-14 years. The mean age of the study population was 12.7±1.3 years (12.8±1.3 for boys and 12.7±1.3 for girls), with no difference between the genders (*p*>0.05). The observed mean height was 1.58±0.09m, significantly higher in boys (mean of 1.59±0.11 boys and 1.56±0.07 in girls; *p*<0.05). The median waist, hip and waist/hip ratio were also significantly higher among male students (*p*>0.05). For the other variables, there were no differences between the genders (Table 1).

**Table 1:** Profile of the study population according to gender.

Variables	Male 265 (52.5%)	Female 240 (47.5%)	Total 505 (100%)	PR (95%CI)	*p*-value
Age (mean±SD)	12.8±1.3	12.7±1.3	12.7±1.3	-	0.471^a^
Age, n (%) (10-14 years)	238 (47.1%)	220 (43.6%)	458 (90.7%)	-	0.001^b^
Age, n (%) (15-16 years)	27 (5.4%)	20 (3.9%)	47 (9.3%)	-	0.775^b^
Height (mean±SD)	1.59±0.1	1.56±0.1	1.58±0.1	-	0.002^a^
Weight, median (IQ)	51.9 (19.7)	49.35 (16.6)	50.3 (18.9)	-	0.335^b^
BMI, median (IQ)	21.05 (5.8)	19.5 (5.2)	19.9 (5.6)	-	0.457^b^
Waist, median (IQ)	71 (12)	66 (11)	67 (12)	-	0.000^b^
Hip, median (IQ)	88 (14)	89 (14)	88 (14)	-	0.001^b^
Waist/hip, median (IQ)	0.80 (0.80)	0.74 (0.70)	0.77 (0.80)	-	<0.001^b^
Overweight, n (%)	49 (18.5%)	43 (18%)	92 (18.2%)	1.03 (0.71-1.50)	
Obesity, n (%)	36 (13.6%)	28 (11.7%)	64 (12.7%)	1.16 (0.73-1.84)	

IQ, interquartile interval; waist/hip, waist-to-hip ratio.

a *t* test for independent samples.

b Mann-Whitney test.

The anthropometric parameter assessment identified body weight alterations in 156 (30.9%) students, of which 92 (18.2%) were overweight and 64 (12.7%) were obese. Weight alterations were slightly more common among boys (32.1%) when compared to girls (29.6%), but with no significant difference between the two groups [PR=1.08 (0.83-1.41)]. Of the observed alterations, overweight was the most frequent one (18.2% of students), affecting 49 (18.5%) boys and 43 (18%) girls [PR=1.03 (0.71-1.50)]. Obesity was found in 64 (12.7%) students, of which 56.3% were boys [PR=1.16 (0.73-1.84)].

When analyzing the dietary habits and daily activities of the schoolchildren, considering the presence of BMI alterations, it was observed that approximately 50% of the children with BMI alteration, i.e., obesity or overweight, admitted consuming sweets and/or soft drinks daily, while daily water intake and the habit of having breakfast, lunch, snacks between meals and dinner daily was more frequently reported by students with normal BMI (Table 2). It was also observed that many students did not perform regular physical activity. Of the 156 students with altered BMI, 104 (66%) admitted not performing any frequent physical activity, 116 (74%) reported spending many of their free hours in activities without energy expenditure and 82 (53%) watched TV for more than three hours a day.

**Table 2:** Profile of the study population regarding dietary and behavioral habits, according to BMI.

Dietary habits	Normal BMI 349 (69.1%)	Altered BMI 156 (30.9%)	PR (95%CI)	*p*-value
*Breakfast daily*				0.183
Yes	158 (31.3%)	60 (11.9%)	0.92	
No	191 (37.8%)	96 (19.0%)	(0.82-1.03)	

*Lunch daily*				0.754
Yes	281 (55.6%)	123 (24.4%)	0.93	
No	68 (13.5%)	33 (6.5%)	(0.83-1.13)	

*Snacks between the main meals*				0.108
Yes	275 (54.5%)	112 (22.2%)	0.88	
No	74 (14.6%)	44 (8.7%)	(0.76-1.03)	

*Dinner daily*				0.670
Yes	286 (56.6%)	125 (24.8%)	0.96	
No	63 (12.5%)	31 (6.1%)	(0.82-1.12)	

*Sweets ≥3 days/week*				0.087
Yes	198 (39.2%)	75 (14.8%)	0.90	
No	151 (29.9%)	81 (16.0%)	(0.8-1.01)	

*Soft drinks ≥3 days/week*				0.186
Yes	196 (38.8%)	77 (15.2%)	0.92	
No	153 (30.3%)	79 (16.6%)	(0.82-1.03)	

*Daily water intake ≥3 glasses*				0.998
Yes	278 (55.1%)	125 (24.7%)	1.01	
No	71 (14.1%)	31 (6.1%)	(0.87-1.17)	

*Physical activity ≥3 days/week*				0.705
Yes	124 (24.5%)	52 (10.3%)	0.97	
No	225 (44.5%)	104 (20.6%)	(0.86-1.1)	

*Electronic device use daily*				0.338
Yes	264 (52.3%)	111 (21.9%)	0.93	
No	85 (16.8%)	45 (8.9%)	(0.81-1.07)	

*Free time without energy expenditure*				0.243
Yes	240 (47.5%)	116 (22.9%)	0.92	
No	109 (21.6%)	40 (7.9%)	(0.81-1.04)	

*TV ≥3hours/day*				0.167
Yes	208 (41.2%)	82 (16.2%)	0.91	
No	141 (27.9%)	74 (14.6%)	(0.81-1.03)	

When evaluating the same variables considering the gender, it was observed that 273 (54.1%) students said they consumed sweets and soft drinks daily, a habit significantly more frequent among girls [PR=0.75 (0.64-0.88) and PR=0.82 (from 0.70 to 0.97), respectively]. The habit of having lunch daily was more often reported by boys [OR=1.11 (1.02-1.22)]. As for the daily water intake and having breakfast, lunch, snacks between meals and dinner daily, were equally reported by students from both groups (Table 3).

**Table 3:** Distribution of food and behavioral variables according to BMI alteration by gender.

	Male 265 (52.5%)				Female 240 (47.5%)		
	Normal BMI 180 (67.9%)	Altered BMI 85 (32.1%)	PR (95%CI)		Normal BMI 169 (70.4%)	Altered BMI 71 (29.6%)	PR (95%CI)
*Breakfast daily*							
Yes	90 (33.9%)	33 (12.4%)	1.15		68 (28.3%)	27 (11.25%)	1.03
No	90 (33.9%)	52 (19.6%)	0.98-1.36		101 (42.0%)	44 (18.3%)	0.87-1.21

*Lunch daily*							
Yes	153 (57.7%)	70 (26.4%)	1.07		128 (53.3%)	53 (22.0%)	1.02
No	27 (10.1%)	15 (50.6%)	0.84-1.36		41 (17.0%)	18 (7.5%)	0.95-1.47

*Snacks between the main meals*							
Yes	142 (53.5%)	63 (23.7%)	1.09		133 (55.4%)	49 (20.4%)	1.18
No	38 (14.3%)	22 (8.3%)	0.88-1.35		36 (15.0%)	22 (9.1%)	0.95-1.47

*Dinner daily*							
Yes	151 (56.9%)	71 (26.7%)	1.01		136 (56.6%)	54 (22.5%)	1.08
No	29 (10.9%)	14 (5.2%)	0.80-1.26		33 (13.7%)	17 (7.0%)	0.87-1.35

*Water intake ≥3 glasses/day*							
Yes	150 (56.6%)	70 (26.4%)	1.02		128 (53.3%)	55 (22.9%)	0.97
No	30 (11.3%)	15 (5.6%)	0.82-1.28		41 (17.0%)	16 (6.6%)	0.81-1.17

*Sweets ≥3 days/week*							
Yes	91 (34.3%)	33 (12.4%)	1.16		107 (44.5%)	42 (17.5%)	1.05
No	89 (33.5%)	52 (19.6%)	0.99-1.37		62 (25.8%)	29 (12.0%)	0.89-1.25

*Soft drinks ≥3 days/week*							
Yes	90 (33.9%)	40 (15.1%)	1.04		106 (44.1%)	37 (15.4%)	1.14
No	90 (33.9%)	45 (16.9%)	0.88-1.23		63 (26.2%)	34 (14.1%)	0.96-1.36

*Physical activities 3 days/week*							
Yes	85 (32.0%)	37 (13.9%)	1.05		39 (16.2%)	15 (6.2%)	1.03
No	95 (35.8%)	48 (18.1%)	0.89-1.24		130 (54.1%)	56 (23.3%)	0.85-1.25

*Electronic device use daily*							
Yes	139 (52.4%)	63 (23.7%)	1.06		125 (52.0%)	48 (20.0%)	1.10
No	41 (15.4%)	22 (8.3%)	0.86-1.30		44 (18.3%)	23 (9.2%)	0.91-1.34

*Free time without energy expenditure*							
Yes	80 (30.1%)	32 (12.0%)	1.09		29 (12.0%)	8 (3.3%)	1.14
No	100 (37.7%)	53 (20.0%)	0.93-1.29		140 (58.3%)	63 (26.2%)	0.94-1.38

*TV ≥3hours/day*							
Yes	105 (39.6%)	43 (16.2%)	1.11		103 (42.9%)	39 (16.2%)	1.08
No	75 (28.3%)	42 (15.8%)	0.93-1.31		66 (27.5%)	32 (13.3%)	0.91-1.28

Only 35% of the study population reported practicing some type of regular physical activity. When assessing the positive responses to this item, the practice of physical activity was most often reported by the group of male students (24% of boys versus 11% of girls). The analysis of the association between this variable and gender showed that boys were two-fold more physically active than girls [PR=2.04 (1.56-2.67)]. As for the variable free hours without energy expenditure, approximately 30% of boys and 40% of girls said they performed no activities with energy expenditure in their free time, with the girls being 32% more inactive than boys [PR=0.68 (0.60-0.76)]. It was observed that boys use more electronic devices (40%) when compared to girls (34%), but there was no significant difference between the genders [PR=1.06 (0.95-1.17)]. Watching TV daily was a very frequent habit. Approximately 60% of all assessed students admitted spending more than three hours a day watching TV, with no difference between the groups [PR=1.06 (0.95-1.17)], suggesting it is a common behavior to the entire assessed population, regardless of gender.

A multiple logistic regression analysis was carried out to assess the influence of lifestyle factors and dietary habits on the likelihood of BMI alterations. The result of the analysis showed that the variables included in the model did not change the likelihood of BMI alteration. The subgroup analysis considering gender also showed no influence of food and behavioral variables on BMI alterations, in male or female students (Table 4).

**Table 4:** Multivariate analysis of the variables BMI alteration and behavioral and dietary habits considering gender.

Variables	Boys 265 (52.5%)				Girls 240 (47.5%)		
	Adjusted PR	95%CI	*p*-value		Adjusted OR	95%CI	*p*-value
Age	0.88	0.72-1.09	0.26		0.81	0.63-1.04	0.10
Breakfast daily	0.62	0.35-1.09	0.10		0.86	0.47-1.57	0.90
Lunch daily	0.82	0.39-1.74	0.61		1.03	0.52-2.02	0.34
Snacks between meals	0.90	0.46-1.77	0.77		0.60	0.31-1.17	0.09
Dinner daily	1.24	0.56-2.75	0.60		0.76	0.38-1.52	0.47
Water intake ≥3 glasses/day	0.84	0.40-1.75	0.64		1.15	0.57-2.30	0.48
Sweets ≥3 days/week	0.64	0.36-1.16	0.14		0.99	0.53-1.84	0.70
Soft drinks ≥3 days/week	1.17	0.65-2.10	0.60		0.69	0.38-1.27	0.21
Physical activity ≥3 days/week	0.90	0.50-1.61	0.72		0.98	0.48-1.20	0.87
Electronic device use daily	0.79	0.41-1.50	0.47		0.72	0.39-1.36	0.70
Free time without energy expenditure	0.96	0.53-1.73	0.90		0.66	0.28-1.58	0.40
TV ≥3hours/day	0.90	0.50-1.62	0.73		0.85	0.47-1.52	0.23

Although significant associations between dietary/behavioral variables and BMI alterations were not observed (Tables 2 and 4), the occurrence of some unhealthy habits and behaviors was confirmed, which were frequent in the studied population, apparently related to the routine life of boys and girls at this age group (Table 4).

## Discussion

The results of this study showed a high prevalence of overweight and obesity in elementary students from public state schools of Ribeirão Preto (SP), providing information on this topic among the population of schoolchildren from the Northwest Region of the state of São Paulo. These data confirm the findings of other studies carried out in other regions^16^^-^ 18 ^,^ 23 ^,^ 24 and also disclosed the presence of an unhealthy lifestyle, such as preference for foods with higher calorie and lower nutritional content, screen time (TV and computers) above that recommended by the WHO and little physical activity.

The prevalence of obesity/overweight found in this study was high and seems to follow the trend observed in the entire country. In 1970, according to the Household Budget Survey (HBS), the rate of excess weight in Brazilian adolescents was 3.7%.^16^ Approximately 30 years later, the prevalence rates were 21.7% (HBS)^16^ and 23.2% (PeNSE).^17^ These data, when evaluated together, show that between 1970 and 2009 in Brazil, the prevalence excess weight in children and adolescents increased four- to five-fold.^16^^,^ 17

Corroborating the data from official documents on the subject, epidemiological studies carried out in different regions of Brazil showed a high prevalence of obesity/overweight in the schoolchildren population, some with values above the national average found by the HBS 2008/2009. Diniz et al., in 2006, studied 4210 students from public schools in Pernambuco and found a prevalence of overweight of 11.5% and 2.4% of obesity.^18^

In another study, published in 2008, the same authors observed overweight in 29.1% of boys and 32.6% of girls^19^ of 694 assessed schoolchildren aged between eight and 11 years, in a municipality in the state of Rio Grande do Sul, prevalence similar to that observed in this study.

Ramos et al. also found high prevalence rates. The authors studied 941 children from public (state and municipal) and private schools, aged 10-14 years, in the city of Campo Grande (MS) and found that 23.1% of the children were overweight.^20^ Souza, when studying 1187 students from public elementary schools in Divinópolis (MG) aged 6-14 years, found 24.4% of the children with obesity and overweight, with 23.5% in girls and 22.6% in boys.^21^ Cabrera et al., also in 2014, observed even higher prevalence rates. When assessing 170 children and 232 adolescents from public schools in the municipality of Nantes (SP), the authors observed weight above the adequate for the age in 30.59% of the assessed students,^22^ a prevalence rate close to that observed in the present study.

Different studies show variable results regarding the prevalence of obesity and overweight among the schoolchildren population.^16^^-^ 19 ^,^ 21 ^-^ 23 Such differences may reflect characteristics related to regional/cultural, genetic and behavioral factors. Additionally, methodological issues, such as study design, different forms of data collection and different parameters used for classification of obesity and overweight may also explain the variability in the study results. However, it is noteworthy the high rates of obesity and overweight observed in the present study and several others.

Excess weight is a chronic condition that acts as a risk factor for other chronic diseases, which, by occurring at such an early stage of life, can predispose very young individuals to preventable diseases and complications. Type 2 diabetes mellitus, systemic arterial hypertension, dyslipidemia, gastroesophageal reflux disease, osteoarthritis, orthopedic and postural problems, certain types of cancers in later phases of life, social adjustment problems and depression are some of the diseases associated with childhood obesity.^24^^,^ 25 A British cohort study, carried out in late 1990, showed that being overweight during childhood resulted in a two-fold increase in the risk of death from ischemic heart disease in adulthood.^26^ Obesity is also related to obstructive sleep apnea-hypopnea syndrome, asthma and exercise intolerance.^25^

The purpose of investigating the life routine of students in the present study was to seek associations between excess weight and behavioral and dietary risk factors that can be prevented or corrected. Apparently contradictory, there was no association between obesity/overweight and the assessed behavioral and dietary factors. Inadequate behaviors and dietary habits were identified in a large proportion of the study population, regardless of the weight.

The high consumption of sweets and soft drinks, low water intake and little physical activity were reported by most students. Additionally, most of the assessed children reported spending long periods of time watching TV or using the computer (screen time), showing that these are very widespread habits and behaviors in children, not specifically restricted to children with altered BMI. The results of a study carried out to evaluate the influence of environmental factors on the lack of physical activity showed that North-American children aged 8-18 years spend an average of 7.5hours/day in mobile phones, electronic games, computers and television, which reduces the available time and motivation to participate in physical activities and active games.^27^ But the lack of physical activity seems to have broader implications. Another recent study showed that sedentary children have reduced physical fitness, with a negative impact on flexibility, strength of several muscle groups and muscle explosion, which are even worse in obese and overweight children.^28^ Therefore, as physical fitness decreases, exercise intolerance increases, further promoting physical inactivity.

However, the assessment of dietary and behavioral variables in the bivariate analyses showed that the use of electronic games is the preferred activity for boys, while girls reported a higher daily consumption of sweets and soft drinks, as well as more time spent with TV and other activities with no energy expenditure, suggesting gender-related preferences.

Several studies have confirmed the increased prevalence of overweight and obesity in schoolchildren of different age groups, regardless of gender. The causes of this increase are not well known, but, as mentioned before, unhealthy dietary habits and lifestyle, as well as genetic, metabolic and environmental factors are involved.^24^^,^ 25 ^,^ 27 Approximately 90% of obesity cases are considered idiopathic and 10% are attributed to hormonal and genetic causes. The latest advances in research indicate the participation of leptin, the hypothalamic melanocortin receptor 4 and mitochondrial uncoupling proteins in the pathogenesis of obesity/overweight, which opens new possibilities for the successful therapeutic management of these conditions,^29^ especially in cases of severe obesity and when measures aimed at changing habits and lifestyle were not successful.

Although the association between overweight and intake of foods with high sugar and fat content and insufficient physical activity is well known,^23^^-^ 25 ^,^ 27 ^,^ 28 this was not observed in this study, probably because they represent widespread practice among the assessed schoolchildren, regardless of body weight. Moreover, the present study showed the association between some dietary habits (eating sweets and soft drinks ≥3 times/week, having lunch daily) and some behaviors of daily living (physical activity ≥3 times/week, free hours without energy expenditure) with gender, which could indicate the need for differentiated preventive interventions for boys and girls.

Childhood obesity is a health problem that has aroused increasing interest among public administrators. Among the goals of the Strategic Action Plan for Confronting Non-communicable Chronic Diseases (NCDs) in Brazil from 2011 to 2022 is the reduction in childhood obesity rates.^30^ Food surveillance, regulation and quality control of food and stimulating physical activity and increased consumption of healthy foods (fruits and vegetables) are initiatives that, above all, ensure better health for all children and adolescents and promote the development of new, more adequate habits.

The results of this study should be interpreted considering some limitations. Although the data show high prevalence of overweight and obesity, in addition to an unhealthy lifestyle and dietary habits in students aged 10-16 years, unrelated to the alterations in body weight, the applicability of these results to other age groups or students from other regions should be considered carefully. The occurrence of possible information bias is also worth mentioning as a study limitation. Data on dietary habits and behaviors were provided by the participants, through interviews. There is a possibility that inaccurate information was provided by the students who were overweight, led to deny or affirm some habits that would cause embarrassment, as mentioned by other authors.^10^

In conclusion, we found a high prevalence of overweight and obesity among schoolchildren aged 10 and 16 years, who attended public state schools in the city of Ribeirão Preto. Although the association with overweight was not found, the weekly time spent with electronic games and TV, as well as the free time devoted to activities that do not involve energy expenditure suggests an inadequate lifestyle for this age group. As a result, low levels of physical activity were identified for the overall assessed population. The same occurred with factors related to dietary habits. The results disclosed the frequent intake of little nutritious food and high intake of foods with little nutritional value (sweets and soft drinks). Although these findings showed no association with obesity or overweight, it is noteworthy the dissemination of these unhealthy habits early in life. Additionally, for the schoolchildren with obesity and overweight, health care and guidelines for the prevention of future disease should be started as soon as possible, respecting the individual differences and preferences.
